# Exact traveling wave solutions for system of nonlinear evolution equations

**DOI:** 10.1186/s40064-016-2219-0

**Published:** 2016-05-26

**Authors:** Kamruzzaman Khan, M. Ali Akbar, Ahmed H. Arnous

**Affiliations:** Department of Mathematics, Pabna University of Science and Technology, Pabna, 6600 Bangladesh; Department of Applied Mathematics, University of Rajshahi, Rajshahi, 6025 Bangladesh; Department of Engineering Mathematics and Physics, Higher Institute of Engineering, El Shorouk, Egypt

**Keywords:** Generalized Kudryashov method, Nonlinear evolution equation, Variant Boussinesq equation, Breaking soliton equations, Exact traveling wave solutions, 35K99, 35P05, 35P99

## Abstract

In this work, recently deduced generalized Kudryashov method is applied to the variant Boussinesq equations, and the (2 + 1)-dimensional breaking soliton equations. As a result a range of qualitative explicit exact traveling wave solutions are deduced for these equations, which motivates us to develop, in the near future, a new approach to obtain unsteady solutions of autonomous nonlinear evolution equations those arise in mathematical physics and engineering fields. It is uncomplicated to extend this method to higher-order nonlinear evolution equations in mathematical physics. And it should be possible to apply the same method to nonlinear evolution equations having more general forms of nonlinearities by utilizing the traveling wave hypothesis.

## Background

The study of autonomous nonlinear evolution equations has a rich and long history, which has continued to attract attention in more recent years. The exact solutions to nonlinear evolution equations are the key tool to understand the various physical phenomena that govern the real world today. Hence searching for exact traveling wave solutions to nonlinear evolution equations plays an important role in the study of nonlinear physical phenomena in many fields such as fluid dynamics, water wave mechanics, meteorology, electromagnetic theory, plasma physics and nonlinear optics.

In the past several decades, there has been significant progress in the development of various methods for finding exact traveling wave solutions to nonlinear evolution equations, such as the Bäcklund transformation (Wahlquist and Estabrook 1973; Luo 2011), the F-expansion method (Liu and Yang 2004; Islam et al. 2014), the tanh method (Wazwaz 2004), the exp-function method (Yusufoglu 2008; Khan and Akbar 2014a), the (G′/G)-expansion method (Wang et al. 2008; Zayed and Al-Joudi 2010; Kim and Sakthivel 2012; Khan and Akbar 2014b; Islam et al. 2013), the functional variable method (Zerarka et al. 2010; Khan and Akbar 2014c; Zayed et al. 2013a), the exp(−Φ(ξ))-expansion method (Khan and Akbar 2014d, 2015), the modified simple equation method (Jawad et al. 2010; Khan and Akbar 2013, 2014e; Ahmed et al. 2013), the homotopy perturbation method (Mohiud-Din 2007; Mohyud-Din and Noor 2009), the Kudryashov method (Kudryashov 2012; Lee and Sakthivel 2013), and the Riccati equation mapping method (Zayed and Arnous 2013a, b).

The aim of this work is to demonstrate the efficiency of the generalized Kudryashov method for finding exact traveling wave solutions transmutable to the solitary wave solutions for system of nonlinear evolution equations. For this purpose, we consider the one dimensional variant Boussinesq equations, and the (2 + 1)-dimensional breaking soliton equations.

## Algorithm of the generalized Kudryashov method

Let us consider the nonlinear evolution equation in two independent variables *x* and *t*:

1 $$ P(u,u_{t} ,u_{x} ,u_{xx} , \ldots ) = 0,\quad x \epsilon {\mathbb{R}},\quad t > 0 $$

where $u = u(x,t)$ is an unknown function, $x$ is the spatial variable and $t$ is the time variable, $P$ is a polynomial in $u$ and its various partial derivatives, in which the highest order derivatives and nonlinear terms are involved.

The main steps of generalized Kudryashov method are as follows (Demiray et al. 2014a, b; Baskonus and Bulut 2015):

### **Step 1:**

The traveling wave variable $\xi = x - \omega \,t$ transforms Eq. (1) into an ordinary differential equation of the form:

2 $$ \varPsi (u,\,u^{{\prime }} ,\,u^{{\prime \prime }} , \ldots ) = 0, $$

where the prime indicates differentiation with respect to $\xi$, and ω ∊ ℝ\{0} is the velocity of the relative wave mode.

Equation (2) may be successively integrated as many times as possible. Remaining to the boundary conditions $u(\xi ) \to 0$ and $\frac{{d^{m} u(\xi )}}{{d\xi^{m} }} \to 0\,(m = 1,\,2,\,3,\, \ldots )$ for $\xi \to \pm \infty$, $\xi = x - \omega \,t$, the constants of integration, if any, should be set to zero (Malfliet and Hereman 1996; Wazwaz 2009).

### **Step 2:**

Suppose that the solution of Eq. (2) has the following form:

3 $$ u(\xi ) = \frac{{\sum\nolimits_{i = 0}^{N} {a_{i} Q^{i} (\xi )} }}{{\sum\nolimits_{j = 0}^{M} {b_{j} Q^{j} (\xi )} }}, $$

where $a_{i} (i = 0,\,1,\,2, \ldots ,N)$ and $b_{j} (j = 0,\,1,\,2, \ldots ,M)$ are constants to be determined afterward such that $a_{N} \ne 0$ and $b_{M} \ne 0$, and $Q = Q(\xi )$ satisfies the following ordinary differential equation:

4 $$ \frac{dQ(\xi )}{d\xi } = Q^{2} (\xi ) - Q(\xi ). $$

The solution of Eq. (4) is as follows:

5 $$ Q(\xi ) = \frac{1}{1 + A\;\exp (\xi )}, $$

where *A* is a constant of integration.

### **Step 3:**

The positive integers *N* and *M* appearing in Eq. (3) can be determined by considering the homogeneous balance between the highest order derivatives and the nonlinear terms come out in Eq. (1) or Eq. (2). Moreover precisely, we define the degree of $u(\xi )$ as $D(u(\xi )) = N - M$ which gives rise to the degree of other expression as follows:

$$ D\left( {\frac{{d^{q} u}}{{d\xi^{q} }}} \right) = N - M + q,\,\,D\left( {u^{p} \left( {\frac{{d^{q} u}}{{d\xi^{q} }}} \right)^{s} } \right) = (N - M)p + s(N - M + q), $$

where $p,\,q,\,s$ are integer numbers.

Therefore, we can find the value of *N* and *M* in Eq. (3).

### **Step 4:**

Substituting Eqs. (3) and (4) into Eq. (2), we obtain a polynomial in $Q^{i - j}$, ($i,j = 0,\,1,\,2, \ldots$). In this polynomial equating the coefficients of all terms of the same powers of *Q* to zero, we obtain a system of algebraic equations which can be solved by using Maple or Mathematica to get the unknown parameters $a_{i} (i = 0,\,1,\,2, \ldots ,N)$, $b_{j} (j = 0,\,1,\,2, \ldots ,M)$, and $\omega$. Consequently, we obtain the exact solutions of Eq. (1).

## Applications

In this section, we will apply the generalized Kudryashov method to construct the exact traveling wave solutions transmutable to the solitary wave solutions for the following two nonlinear evolution equations:

### *Example 1. The variant Boussinesq equations:*

In this subsection, we will apply the generalized Kudryashov method to find the exact solutions and then the solitary wave solutions to the variant Boussinesq equations (Wang et al. 2008; Khan and Akbar 2013, 2014b) in the form,

6 $$ \begin{aligned} u_{t} + H_{x} + u\,u_{x} & = 0, \\ H_{t} + \left( {u\,H} \right)_{x} + u_{x\,x\,x} & = 0, \\ \end{aligned} $$

which was derived by Sachs in the year 1988 (Sachs 1988) as a model for water waves (Guo et al. 2015), where $u(x,t)$ is the velocity, $H(x,t)$ is the total bottom depth of the region occupied by the fluid and the subscripts denote the partial derivatives. The Boussinesq equation is a celebrated model of long water wave of moderate amplitude, which describes one dimensional, and weakly nonlinear internal wave which develops at the boundary between two immiscible fluids. Besides, the equation is a simplified model of the atmospheric movement equation which is applicable to mesoscale and quasi-incompressible fluid movement, which means important physical applications in hydrodynamics. The Boussinesq equation also is of considerable mathematic interests because of its rich mathematical structures (Guo et al. 2015).

The traveling wave transformation is defined by,

7 $$ u(\xi ) = u(x,t),\,\,\,H(\xi ) = H(x,t),\,\,\,\,\xi = x - \omega \,t. $$

Using traveling wave Eqs. (7), (6) transform into the following ordinary differential equations:

8 $$ - \omega \,u^{{\prime }} + H^{{\prime }} + uu^{{\prime }} = 0, $$

9 $$ - \omega H^{\prime } + (uH)^{\prime } + u^{\prime\prime\prime} = 0. $$

Integrating Eqs. (8) and (9) with respect to *ξ*, choosing the constant of integration as zero (under the boundary conditions described in “Algorithm of the generalized Kudryashov method” section (Step 1) and using similar boundary conditions for H(ξ)), we obtain the following ordinary differential equations respectively:

10 $$ - \omega u + H + \frac{1}{2}u^{2} = 0, $$

11 $$ - \omega H + uH + u^{{\prime \prime }} = 0. $$

From Eq. (10), we get

12 $$ H = \omega \,u - \frac{1}{2}u^{2} . $$

Substituting Eq. (12) into Eq. (11), we obtain

13 $$ u^{{\prime \prime }} - \omega^{2} u + \frac{3}{2}\omega \,u^{2} - \frac{1}{2}u^{3} = 0. $$

Now balancing the highest order derivative $u^{{\prime \prime }}$ and nonlinear term $u^{3}$, we get $3N - 3M = N - M + 2$ or equivalent to $N = M + 1$.

Setting $M = 1$, we obtain $N = 2$. Therefore, Eq. (3) reduces to

14 $$ u(\xi ) = \frac{{a_{0} + a_{1} Q + a_{2} Q^{2} }}{{b_{0} + b_{1} Q}}. $$

Substituting Eq. (14) along with Eq. (4) into Eq. (13), we get a polynomial of $Q^{k}$, ($k = 0,\,1,\,2, \ldots$). Equating the coefficients of this polynomial of the same powers of Q to zero, we obtain a system of algebraic equations. This system of equations yields the values for $\omega ,a_{0} ,a_{1} ,a_{2} ,b_{0}$ and $b_{1}$.

$$ {\text{Set }}1:\quad \omega = \pm 2,\,a_{0} = 0,\,a_{1} = 0,\,a_{2} = \pm 2b_{1} ,\,b_{0} = - 0.50\,b_{1} . $$

$$ {\text{Set }}2:\quad \omega = \pm 1,\,a_{0} = 0,\,\,a_{2} = 0,\,b_{0} = - \,b_{1} \pm \frac{1}{2}a_{1} . $$

$$ {\text{Set }}3:\quad \omega = \pm 1,\,a_{0} = 0\,,\,a_{1} = \pm 2b_{0} ,a_{2} = \pm 2b_{1} . $$

$$ {\text{Set }}4:\quad \omega = \mp 1,\,a_{0} = \mp 2b_{0} \,,\,a_{1} = \mp \left( {2b_{1} - 2b_{0} } \right),a_{2} = \pm 2b_{1} . $$

$$ {\text{Set }}5:\quad \omega = \mp 2,\,a_{0} = \pm 2b_{1} \,,\,a_{1} = \mp 4b_{1} ,a_{2} = \pm 2b_{1} ,\,b_{0} = - \frac{1}{2}b_{1} . $$

$$ {\text{Set }}6:\quad \omega = \pm I\sqrt 2 ,\,a_{0} = \mp \frac{{Ib_{1} }}{\sqrt 2 },a_{1} = \mp 2b_{1} \pm I\sqrt 2 b_{1} ,a_{2} = \pm 2b_{1} ,b_{0} = - \frac{{b_{1} }}{2}. $$

Set 1 corresponds to the following solutions for the variant Boussinesq equations:

15 $$ u(x,t) = \mp \frac{4}{{A^{2} \;\exp (2x \mp 4t) - 1}}, $$

16 $$ H(x,t) = - \frac{{8A^{2} \;\exp (2x \mp 4t)}}{{\left( {A^{2} \;\exp (2x \mp 4t) - 1} \right)^{2} }}. $$

Set 2 corresponds to the following solutions for the variant Boussinesq equations:

17 $$ u(x,t) = \pm \frac{{2\,a_{1} }}{{Aa_{1} \;\exp (x \, \mp t) \mp 2b_{1} A\;\exp (x \mp t) + a_{1} }}, $$

18 $$ H(x,t) = \frac{{2Aa_{1} (a_{1} \mp 2b_{1} )\;\exp (x \mp t)}}{{\left( {Aa_{1} \;\exp (x \mp t) \mp 2b_{1} A\;\exp (x \mp t) + a_{1} } \right)^{2} }}. $$

Set 3 corresponds to the following solutions for the variant Boussinesq equations:

19 $$ u(x,t) = \pm \frac{2}{1 + A\;\exp (x \mp t)}, $$

20 $$ H(x,t) = \frac{2A\;\exp (x \mp t)}{{\left( {1 + A\;\exp (x \mp t)} \right)^{2} }}. $$

Set 4 corresponds to the following solutions for the variant Boussinesq equations:

21 $$ u(x,t) = \mp \frac{2A\;\exp (x \pm t)}{1 + A\;\exp (x \pm t)}, $$

22 $$ H(x,t) = \frac{2A\;\exp (x \pm t)}{{\left( {1 + A\;\exp (x \pm t)} \right)^{2} }}. $$

Set 5 corresponds to the following solutions for the variant Boussinesq equations:

23 $$ u(x,t) = \pm \frac{{4A^{2} \;\exp (2x \pm 4t)}}{{1 - A^{2} \;\exp (2x \pm 4t)}}, $$

24 $$ H(x,t) = - \frac{{8A^{2} \;\exp (2x \pm 4t)}}{{\left( {A^{2} \;\exp (2x \pm 4t) - 1} \right)^{2} }}. $$

Set 6 corresponds to the following solutions for the variant Boussinesq equations:

25 $$ u(x,t) = \pm \left( {I\sqrt 2 \mp \frac{4A\;\exp (x \mp I\sqrt 2 \,t)}{{A^{2} \;\exp (2x \mp 2I\sqrt 2 t) - 1}}} \right), $$

26 $$ H(x,t) = - \left( {1 + \frac{{8A^{2} \;\exp (2x \mp 2I\sqrt 2 \,t)}}{{\left( {A^{2} \;\exp (2x \mp 2I\sqrt 2 t) - 1} \right)^{2} }}} \right). $$

### *Remark*

**Fig. 1:**
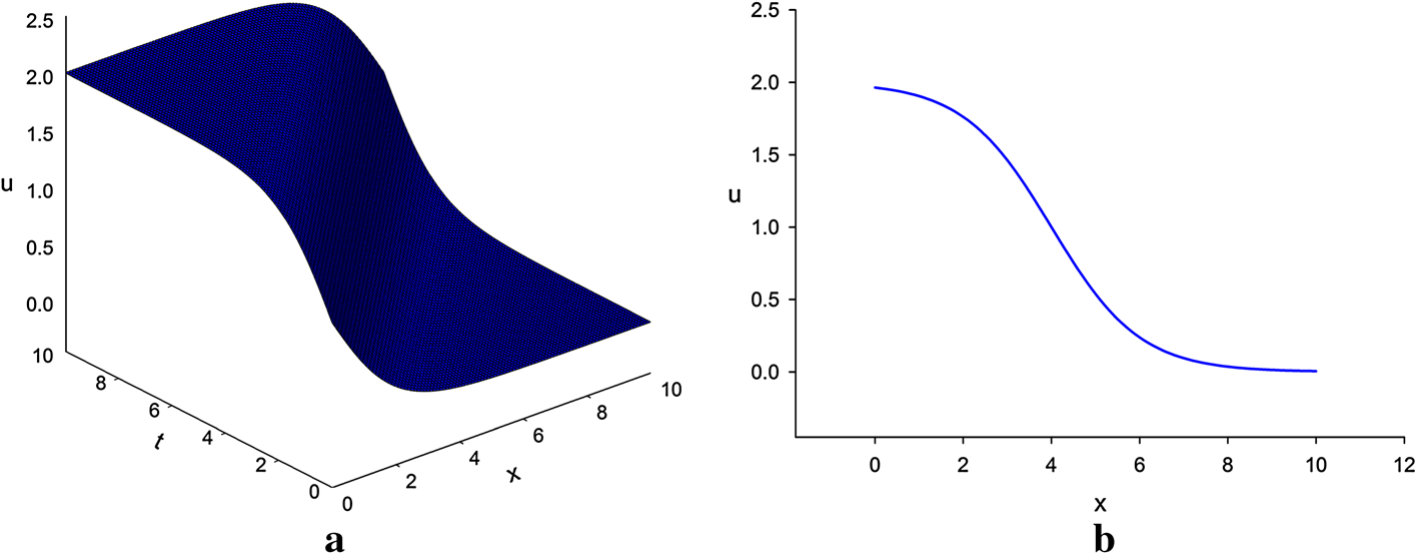
Kink profile of variant Boussinesq equations for A = 1 [only shows the shape of the graph described by Eq. (27)]. **a** The 3D profile, and **b** the 2D profile for t = 4

**Fig. 2:**
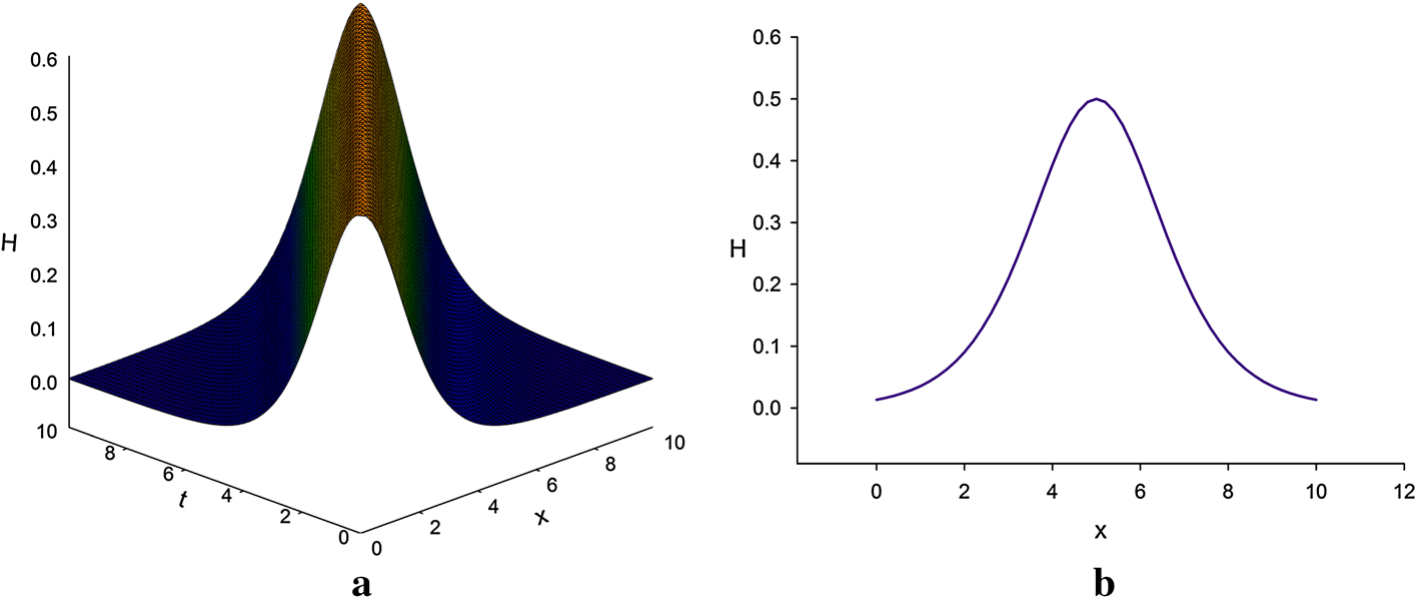
Bell-shaped bottom depth profile of variant Boussinesq equations for A = 1 [only shows the shape of the graph described by Eq. (28)]. **a** The 3D profile, and **b** the 2D profile for t = 5

The bottom depth *H*(*x,t*) must be a non-negative and real physical quantity. Solutions (17)–(22) of the variant Boussinesq equations are significant both mathematically and physically for their positive sign for *H*(*x, t*). Besides solutions (15) and (23) are valid mathematically and physically for their positive and negative signs for *u*(*x, t*) but their corresponding solutions (16) and (24) are valid only mathematically. Solutions (25) and (26) are complex solutions, therefore although they are logically true but they have no physical significance (Figs. 1, 2).

We can obtain some traveling wave solutions since A is an arbitrary constant of integration, for example.

If we put *A* = 1 into Eqs. (19) and (20) and considering *u*(*x*, *t*) > 0 as well as a wave moving to the right, i.e., in the positive direction of *x*-axis, we obtain

27 $$ u(x,\,t) = 1 - \tanh \left( {\frac{1}{2}\left( {x - t} \right)} \right), $$

28 $$ H(x,\,t) = \frac{1}{2}\sec \;h^{2} \left( {\frac{1}{2}\left( {x - t} \right)} \right). $$

### *Example 2: The (2* *+* *1)-dimensional breaking soliton equations*:

Now, we will investigate explicit exact traveling wave solutions of the following (2 + 1)-dimensional breaking soliton equations (Zayed et al. 2013b):

29 $$ u_{t} + \alpha \,u_{xxy} + 4\alpha \left( {uv} \right)_{x} = 0, $$

30 $$ u_{y} = v_{x} , $$

where α is a nonzero constant. Equations (29) and (30) describe the (2 + 1)-dimensional interaction of a Riemann wave propagation along the y-axis with a long wave propagated along the x-axis.

Applying the traveling wave variable $\xi = x + y - \omega \,t$ and proceeding as before, we obtain

31 $$ - \omega \,u^{\prime } + \alpha \,u^{\prime\prime\prime} + 4\alpha \left( {uv} \right)^{\prime } = 0, $$

32 $$ u^{{\prime }} = v^{{\prime }} . $$

Integrating Eq. (32), we obtain

33 $$ v = u, $$

choosing constant of integration as zero under the boundary conditions elucidated in “Algorithm of the generalized Kudryashov method” section (Step 1) and similar boundary conditions for v(ξ).

Substituting Eq. (33) into Eq. (31) and integrating, we get

34 $$ \alpha \,u^{{\prime \prime }} - \omega \,u + 4\alpha \,u^{2} = 0, $$

choosing constant of integration to zero under the boundary conditions mentioned in “Algorithm of the generalized Kudryashov method” section (Step 1).

Considering the homogeneous balance between $u^{{\prime \prime }}$ and $u^{2}$ in Eq. (34), we obtain $N = M + 2$.

Setting $M = 1$, we obtain $N = 3$. Therefore, Eq. (3) takes the form

35 $$ u(\xi ) = \frac{{a_{0} + a_{1} Q + a_{2} Q^{2} + a_{3} Q^{3} }}{{b_{0} + b_{1} Q}}. $$

Substituting Eq. (35) along with Eq. (4) into Eq. (34), we get a polynomial of $Q^{k}$, ($k = 0,\,1,\,2, \ldots$). Equating the coefficients of the polynomial of the same powers of Q to zero, we obtain a system of algebraic equations. This system of equations yields the values for $\omega ,a_{0} ,a_{1} ,a_{2} ,b_{0}$ and $b_{1}$.

$$ {\mathbf{Set}} \, {\mathbf{1}}:\omega = - \alpha ,\,a_{0} = - \frac{1}{4}\,b_{0} ,\,a_{1} = \frac{3}{2}b_{0} - \frac{1}{4}\,b_{1} ,\,a_{2} = \frac{3}{2}\,\left( {b_{1} - b_{0} } \right),\,a_{3} = - \frac{3}{2}b_{1} $$

$$ {\mathbf{Set}} \, {\mathbf{2}}:\omega = \alpha ,\,a_{0} = 0,\,a_{2} = \frac{3}{2}b_{1} - a_{1} ,\,a_{3} = - \frac{3}{2}b_{1} ,\,b_{0} = \frac{2}{3}a_{1} $$

Set 1 corresponds to the following solutions for the breaking soliton equations:

36 $$ u\left( {x,y,t} \right) = v\left( {x,y,t} \right) = \frac{{4A\;\exp \left( {x + y + \alpha \,t} \right) - A^{2} \;\exp \left( {2x + 2y + 2\alpha \,t} \right) - 1}}{{4\left( {1 + A\;\exp \left( {x + y + \alpha \,t} \right)} \right)^{2} }}. $$

If we set A = 1, then Eq. (36) transforms to

37 $$ u\left( {x,y,t} \right) = v\left( {x,y,t} \right) = \frac{1}{8}\left( {3\sec \;h^{2} \left( {\frac{1}{2}\left( {x + y + \alpha \,t} \right)} \right) - 2} \right). $$

Again, if we set A = −1, then Eq. (36) becomes

38 $$ u\left( {x,y,t} \right) = v\left( {x,y,t} \right) = - \frac{1}{8}\left( {3\csc \,h^{2} \left( {\frac{1}{2}\left( {x + y + \alpha \,t} \right)} \right) + 2} \right). $$

Set 2 corresponds to the following solutions for the breaking soliton equations:

39 $$ u\left( {x,y,t} \right) = v\left( {x,y,t} \right) = \frac{{3A\;\exp \left( {x + y - \alpha \,t} \right)}}{{2\left( {1 + A\;\exp \left( {x + y - \alpha \,t} \right)} \right)^{2} }}. $$

If we set A = 1, then Eq. (39) becomes

40 $$ u\left( {x,y,t} \right) = v\left( {x,y,t} \right) = \frac{3}{8}\sec \,h^{2} \left( {\frac{1}{2}\left( {x + y - \alpha \,t} \right)} \right). $$

**Fig. 3:**
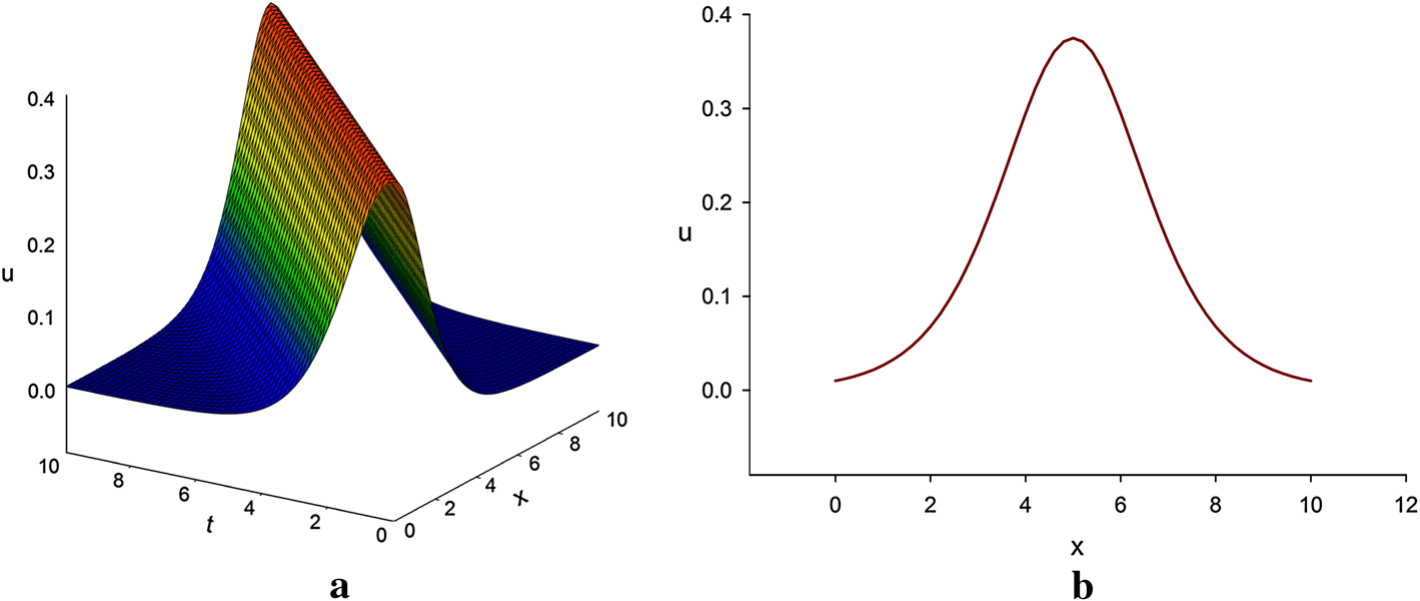
Bell-shaped profile of breaking soliton equation [only shows the shape of the graph described by Eq. (40)]. **a** The 3D profile for *y* = 0 and *α* = 1 and **b** the correspondent 2D profile for *t* = 2.5

Again, if we set A = −1, then Eq. (39) becomes (Fig. 3)

41 $$ u\left( {x,y,t} \right) = v\left( {x,y,t} \right) = - \frac{3}{8}\csc \,h^{2} \left( {\frac{1}{2}\left( {x + y - \alpha \,t} \right)} \right). $$

### Comparisons

1. Khan and Akbar (2013) studied the variant Boussinesq equations by means of the modified simple equation method and found only four solutions (see “Appendix 1”). On the other hand, by using the generalized Kudryashov method we have found twelve solutions. Moreover, if we put *A* = 1 into our solutions Eqs. (19) and (20), then these solutions coincide with the solutions (42) and (44) obtained by Khan and Akbar (2013) for the value of $\omega = - 1$ and $\omega = 1$ (see “Appendix 1”). Similarly, if we put A = −1, then our solutions Eqs. (19) and (20) coincide with the solutions (43) and (45) for the values of $\omega = - 1$ and $\omega = 1$ obtained by Khan and Akbar (2013).

The remaining solutions of Khan and Akbar (2013) given in “Appendix 1” are obtained changing ξ by −ξ in our Eq. (19). Note that all the solutions obtained here are also valid when one replaces the traveling wave variable ξ by −ξ.

2. Zayed et al. (2013b) investigated exact traveling wave solutions to the (2 + 1)-dimensional breaking soliton equation by means of the functional variable method and found only one solution (see “Appendix 2”). On the other hand, by using the generalized Kudryashov method we found four solutions from which one of our solutions coincides with the solution of Zayed et al. If we set $c = \alpha$ into the solution (46) (see “Appendix 2”) obtained by Zayed et al. (2013b), then our solution (41) coincides with that solution.

From the above discussion, we conclude that the generalized Kudryashov method is a more reliable technique, in principle, than the modified simple equation method and the functional variable method.

## Conclusions

In this article, we have successfully presented a mathematical tool named the generalized Kudryashov method for finding exact traveling wave solutions to the variant Boussinesq equations, and the (2 + 1)-dimensional breaking soliton equations. The obtained results will serve as a very important milestone in the study of plasma physics and water waves phenomena. We also have demonstrated that the generalized Kudryashov method is an effective tool for obtaining exact analytical solutions for large classes of system of nonlinear evolution equations.

## Appendix 1

Khan and Akbar (2013) found the following exact traveling wave solutions of the variant Boussinesq equations by using the modified simple equation method:

42 $$ u_{1,\,2} \left( {x,\,t} \right) = \omega \,\left( {1 \pm \;\tanh \left( {\frac{\omega }{2}\left( {x - \omega \,t} \right)} \right)} \right), $$

43 $$ u_{3,\,4} \left( {x,\,t} \right) = \omega \,\left( {1 \pm \;\coth \left( {\frac{\omega }{2}\left( {x - \omega \,t} \right)} \right)} \right), $$

44 $$ H_{1} \left( {x,\,t} \right) = \frac{{\omega^{2} }}{2}\sec \;h^{2} \left( {\frac{\omega }{2}\left( {x - \omega \,t} \right)} \right), $$

45 $$ H_{2} \left( {x,\,t} \right) = - \frac{{\omega^{2} }}{2}\cos ech^{2} \left( {\frac{\omega }{2}\left( {x - \omega \,t} \right)} \right). $$

## Appendix 2

Zayed et al. (2013b) found the following exact traveling wave solution to the (2 + 1)-dimensional Breaking Soliton Equation by means of functional variable method:

46 $$ u\left( {x,y,t} \right) = v\left( {x,y,t} \right) = \frac{ - 3c}{8\alpha }\csc \,h^{2} \left( {\frac{1}{2}\sqrt {\frac{c}{\alpha }} \left( {x + y - c\,t} \right)} \right). $$
